# Supplementary material for: Host-Dependent Variation in Tetranychus urticae Fitness and Microbiota Composition Across Strawberry Cultivars
Source: Insects. 2025 Jul 25;16(8):767. doi: 10.3390/insects16080767 (PMC12386256; doi:10.3390/insects16080767)
Supplement: Supplementary file 1 [file insects-16-00767-s001.zip › insects-3698047-supplementary.pdf]

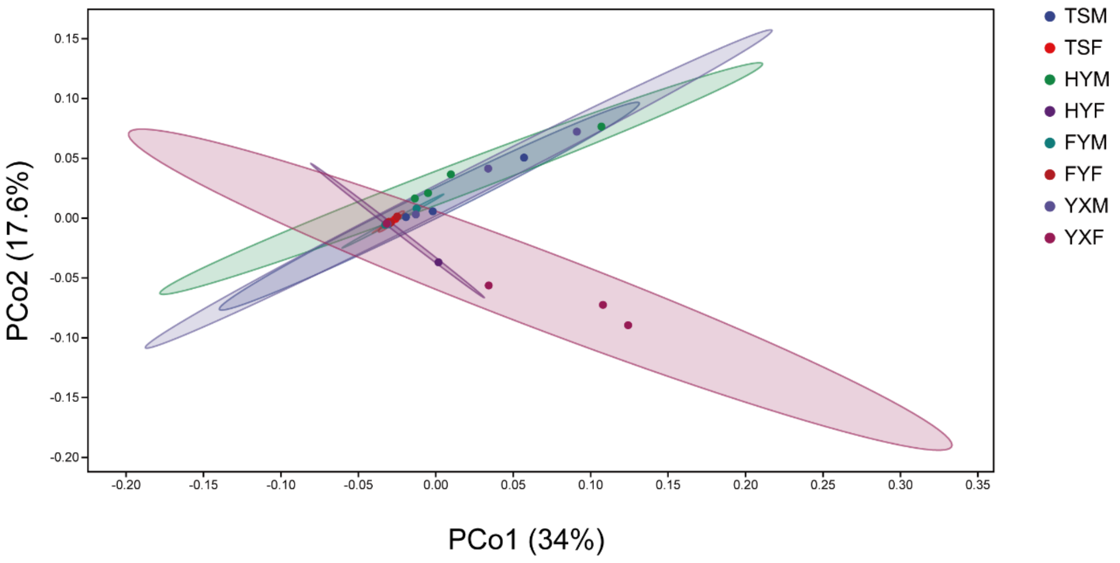

Figure S1. Beta diversity analysis of bacterial communities of *Tetranychus urticae* reared on four Chinese strawberry cultivars: Tianshi (TS), Hongyan (HY), Fenyu (FY), and Yuexiu (YX), by 16s rRNA gene sequencing, the results of PCoA analysis was shown.

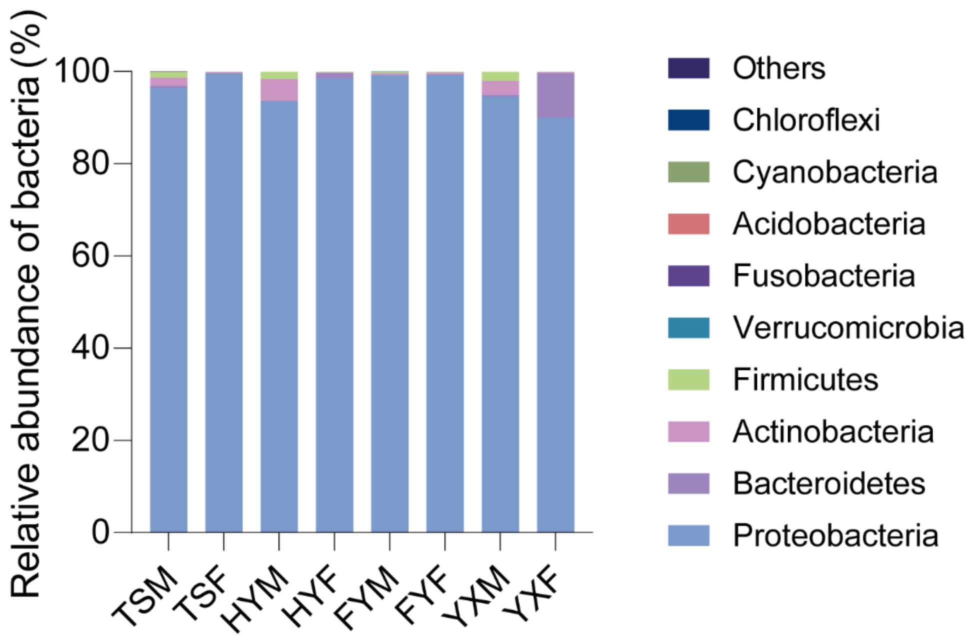

Figure S2. Relative abundance of top 10 bacterial phylum in *Tetranychus urticae* 1-day-old male/female adults on 4 Chinese strawberry cultivars Tianshi (TS), Hongyan (HY), Ningyu (NY), Fenyu (FY) and Yuexiu (YX), by full-length 16s rRNA gene sequencing. In the group labels, the third letter denotes the sex of the mites: 'F' for female and 'M' for male.

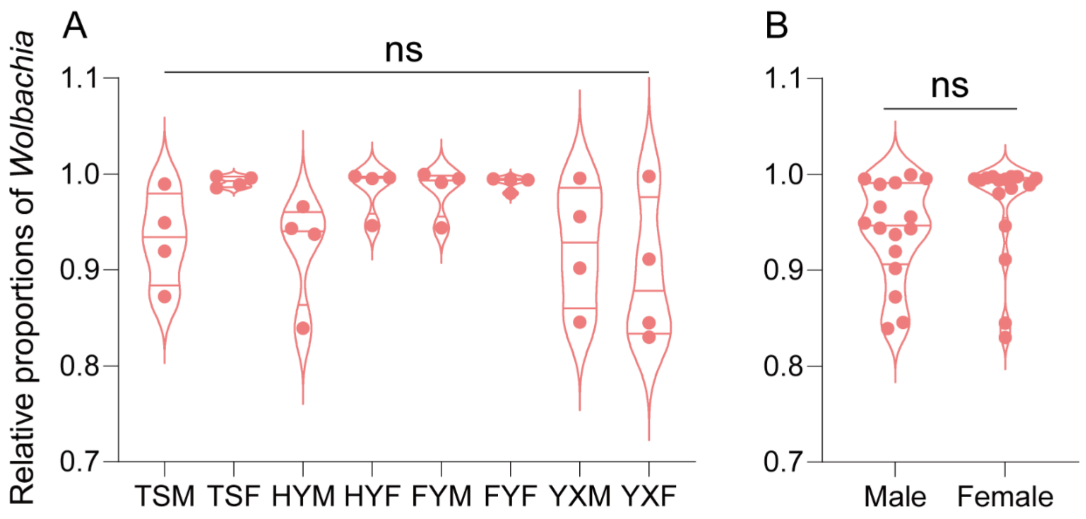

Figure S3. *Wolbachia* proportions in bacterial communities in 1-day-old male and female *Tetranychus urticae* adults reared on four Chinese strawberry cultivars: Tianshi (TS), Hongyan (HY), Fenyu (FY), and Yuexiu (YX). In the group labels, the third letter denotes the sex of the mites: 'F' for female and 'M' for male. ns: no significant difference.

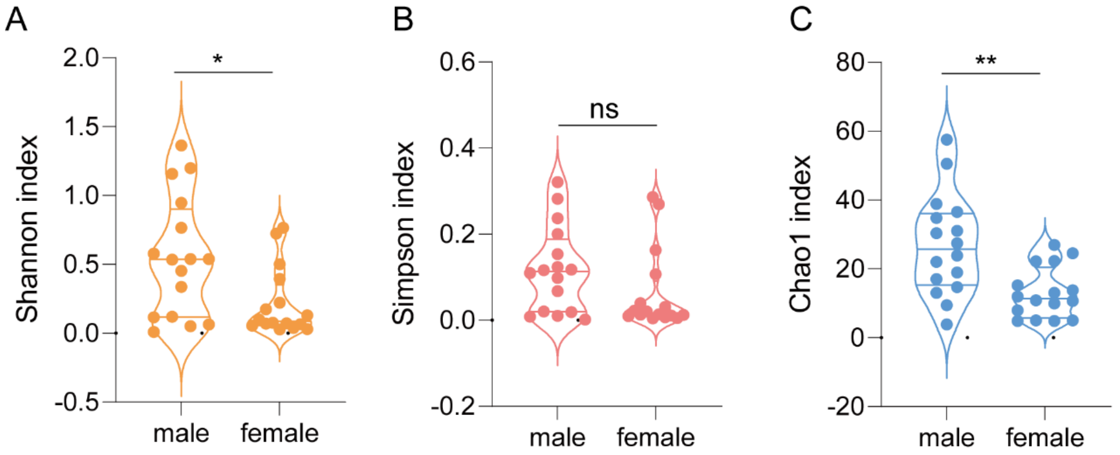

Figure S4. Alpha diversity indices of bacterial communities in 1-day-old male and female *Tetranychus urticae* adults reared on four Chinese strawberry cultivars: Tianshi (TS), Hongyan (HY), Fenyu (FY), and Yuexiu (YX). The diversity metrics presented include Shannon (A), Simpson (B), and Chao1 (C) indices. In the group labels, the third letter denotes the sex of the mites: 'F' for female and 'M' for male.
